# Severe and critical COVID-19 in a tertiary center in Colombia, a retrospective cross-sectional study

**DOI:** 10.1186/s12879-022-07246-0

**Published:** 2022-03-12

**Authors:** Deving Arias Ramos, Diana Lizbeth Restrepo Rueda, Erika Vanessa Rios Quintero, Juan Camilo Olaya Gómez, Isabella Cortés Bonilla

**Affiliations:** 1 Internal Medicine Physician, CAC Santa Bárbara, Palmira, Colombia; 2 Respiratory Therapist, CAC Santa Bárbara, Palmira, Colombia; 3 General Physician, CAC Santa Bárbara, Palmira, Colombia; 4 Grupo de Investigación en Medicina Interna, CAC Santa Bárbara, Palmira, Colombia; 5 Grupo de Investigación en Medicina Interna, Universidad Tecnológica de Pereira, Pereira, Colombia

**Keywords:** COVID-19, ARDS, Invasive mechanical ventilation, In-hospital mortality, Latin America

## Abstract

**Background:**

Colombia has been one of the Latin American countries seriously affected by the covid-19 pandemic. Risk factors for severe disease and death in COVID 19 have been described across the world. Here we report the outcomes, clinical characteristics and risk factors for invasive mechanical ventilation and in-hospital death in a tertiary center in Palmira, Colombia.

**Methods:**

This was a retrospective cross-sectional study involving one single center in Palmira, Colombia. People hospitalized with severe and critical covid-19, during the first pandemic wave, were included. The clinical characteristics and risk factors for in-hospital mortality and invasive mechanical ventilation were mean to be stablished by using a logistic regression analysis.

**Results:**

One hundred and fifty-eight patients were analyzed. Most patients were male (70%) with a mean age of 63 years, invasive mechanical ventilation was provided to 39%, in-hospital mortality was 36%, mainly caused by refractory hypoxemia and septic shock, admission to intensive care was as high as 65%. The logistic regression analysis showed that the risk factors for in-hospital mortality were elevated levels of lactic dehydrogenase and high-sensitivity troponin I, acute renal failure, COPD, and > 10 points on the MuLBSTA score. The risk factors for invasive mechanical ventilation were high levels of C-reactive protein and very low lymphocyte counts, a PaO2/FiO_2_ < 70 and some clinical scores like CURB65, NEWS 2, and PSI/PORT.

**Conclusions:**

During the first pandemic wave in Colombia, for the experience of a tertiary center with a mainly elderly population, a high prevalence of severe ARDS was found, high requirement of intensive care, invasive ventilatory support, bacterial sepsis and an elevated mortality rate were found. The risk factors for in-hospital death and invasive mechanical ventilation were stablished.

**Supplementary Information:**

The online version contains supplementary material available at 10.1186/s12879-022-07246-0.

## Background

The ‘coronavirus disease 2019’ (COVID-19), was declared a pandemic by the WHO on March 11, 2020, with Severe acute respiratory syndrome coronavirus 2 (SARS-CoV-2) as the causative agent [1–3]. The first case of SARS-CoV-2 infection in Latin America (LA) was reported on February 26, 2020 in Brazil and, on March 6, the first COVID-19 case was diagnosed in Colombia in a person who traveled from Italy [3, 4].

SARS-CoV-2 can cause a serious life-threatening disease [1, 2]. The spectrum of disease severity is broad and ranges from asymptomatic and mild illness to severe and critical illness [1]. The disease mainly affects adults and the elderly, most cases are mild (81%), but 14% had severe disease, which can quickly lead to critical illness characterized by presence of acute respiratory distress syndrome (ARDS), sepsis, or septic shock [5]. The clinical manifestations tend to be severe in older men (> 60 years old) with comorbidities [1, 6].

High mortality rate from severe and critical COVID-19 have been recorded worldwide [7]. Several risk factors have been established for severe and critical illness. The mortality rate reported depends on the population analyzed in each study [1, 8]. A descriptive study of 138 patients in a single center in Wuhan, China showed that 26% of patients were admitted to intensive care and mortality was 4.3% [9]. In Belgium, Van Halem et al. reported a fatality rate of 25% during the first weeks of the epidemic, mainly associated with risk factors such as advanced age, kidney failure, elevated lactate levels, lactate dehydrogenase (LDH) levels, and thrombocytopenia [7]. High mortality rates have been reported in studies in Latin American and European patients with any medical condition, mostly in older adults and men [8]. ARDS appears to be one of the manifestations of COVID-19 with the highest mortality burden. It has been known since the pre-COVID-19 era that ARDS carries significant mortality, as high as 46.1% for those with severe ARDS [10]. Steroids have shown to be a cost-effective pharmacological intervention to reduce mortality and possibly days of invasive mechanical ventilation (IMV) in severe a critical COVID-19 [11, 12].

The aim of the study was to describe the clinical characteristics of patients hospitalized for severe and critical COVID-19 attended in a tertiary center from Colombia, and to establish the risk factors for the requirement of IMV and in-hospital mortality. This was a single center study, developed in a city called Palmira, in Colombia. Palmira is a main agricultural city in Colombia, with a population of over 300,000 inhabitants.

## Methods

### Study design and data collection

This was a retrospective cross-sectional study conducted at the Santa Bárbara Clinic, an over 100 beds tertiary care center located in the city of Palmira, Colombia. All patients aged 18 years or older admitted for confirmed severe and critical COVID-19 were included in the study. The study was approved by the Ethics Committee of Santa Bárbara Clinic. The requirement for informed consent was waived because of the retrospective nature of the study. The study period was from March 20, 2020, to November 11, 2020. In this period, the first wave of the pandemic occurred in Colombia. Patients were excluded if they had mild to moderate COVID-19, if they were hospitalized in another center for > 7 days (and then transferred to our center), if they did not receive dexamethasone and, if patients consciously refused receiving IMV, while they needed it, and died.

The electronic medical records were reviewed. We collected data on patient clinical characteristics, comorbidities, symptoms and laboratory tests at hospital admission. A logistic regression analysis was constructed. Risk factors for in-hospital mortality and IMV were stablished.

### Case definition

- Severe COVID-19 was defined as any adult patient with COVID-19 presenting with clinical signs of pneumonia (fever, cough, dyspnea, fast breathing) and one of the following: respiratory rate > 30 breaths/min; severe respiratory distress; or oxygen saturation < 90% on room air [5, 6, 13].
- Critical COVID-19 was defined as any adult patient with COVID-19 presenting with clinical signs of pneumonia and one of the following: presence of acute respiratory distress syndrome or respiratory failure requiring ventilation and/or septic shock [5, 6, 13].
- ARDS was defined based on the Berlin definition for patients undergoing mechanical ventilation [14].
- Sepsis was defined according to the The Third International Consensus Definitions for Sepsis and Septic Shock (Sepsis-3) [15]

### Intensive care unit admission

Patients were admitted to the intensive care unit if they met the following criteria:

- Acute respiratory distress syndrome or acute respiratory failure requiring invasive mechanical ventilation.
- Septic shock.
- Severe Covid-19 requiring support with high flow nasal oxygen (HFNO) or non-invasive mechanical ventilation (NIV).

Before intensive care admission, critical care capacity and the prognosis of each patient according to medical criteria were also considered. Barthel Index for Activities of Daily Living (ADL), Charlson Comorbidity Index (CCI) and the Clinical Frailty Scale were used for decision making in elderly patients. The wishes of each patient and of each one's family were considered, in such a way that end-of-life care was determined and acceptance of orotracheal intubation was established before admission to the ICU.

### Orotracheal intubation vs non-invasive ventilatory support

- Orotracheal intubation was considered as the initial approach for patients with acute respiratory failure, mental status changes, shock requiring vasopressors or multi-organ failure accompanying hypoxemia.
- High flow nasal oxygen (HFNO) was considered for all cooperative patients with the ability to protect the airway, PCO_2_ ≤ 45 or pH > 7.30, requiring FiO_2_ > 50% without high work of breathing. The ROX index was evaluated, orotracheal intubation was considered when it was < 3.8.
- Non-invasive ventilatory support was considered for patients with severe COVID-19 with congestive heart failure (CHF), Obstructive sleep apnea (OSA), Chronic obstructive pulmonary disease (COPD) exacerbations and Obesity hypoventilation syndrome (OHS) presenting with PCO_2_ > 45 or pH ≤ 7.35 and high work of breathing.

### Laboratory methods

Specimens analyzed included mainly blood and respiratory samples. Laboratory tests included complete blood cell count, liver function tests, renal function tests, ferritin levels, serum lactate dehydrogenase (LDH) levels and D-dimer levels. Serum D-dimer levels were established using the ELFA technique (Enzyme Linked Fluorescent Assay) (BioMérieux). Chest X-rays and chest CT scans were performed when appropriate. To confirm the diagnosis of COVID-19, Real-time reverse transcriptase-polymerase chain reaction (RT-PCR) for SARS-CoV-2 in a respiratory sample was used. Patients with a positive test for SARS-CoV-2 antigen in a nasopharyngeal sample could also be included. ABBOTT's COVID-19 Ag Rapid Test was used.

### Microbiological studies

Blood cultures and respiratory tract samples (from endotracheal aspirate and orotracheal tube) were taken when appropriate, mainly for evaluation of bacterial co-infections in COVID-19 patients admitted to ICU. Antimicrobial susceptibility testing was performed according to Clinical Laboratory Standards Institute (CLSI) recommendations, Vitek 2 system (bioMérieux SA) was used. All patients requiring IMV with suspicion of bacterial pneumonia were eligible for testing with FilmArray Pneumonia Panel (FAPP; bioMérieux, France).

### Statistical analysis

We used descriptive statistics to analyze data. Normality was evaluated using the Kolmogorov Smirnov Test. Continuous variables were presented as mean ± standard deviation or, as medians and interquartile ranges (IQR) when appropriate. Categorical variables were presented as frequency and percentage. Categorical variables were analyzed using the Chi-square test or the Fisher Exact test when appropriate. For continuous data, the assumptions of normality were verified and for those that fulfilled them, Student's T tests were performed. Non-parametric tests were used for those that did not fulfill the assumptions of normality.

A logistic regression analysis was constructed. All associations were considered significant for a value of p < 0.05. IBM SPSS Statistics software version 20 was used for all statistical analyses.

## Results

**Fig. 1:**
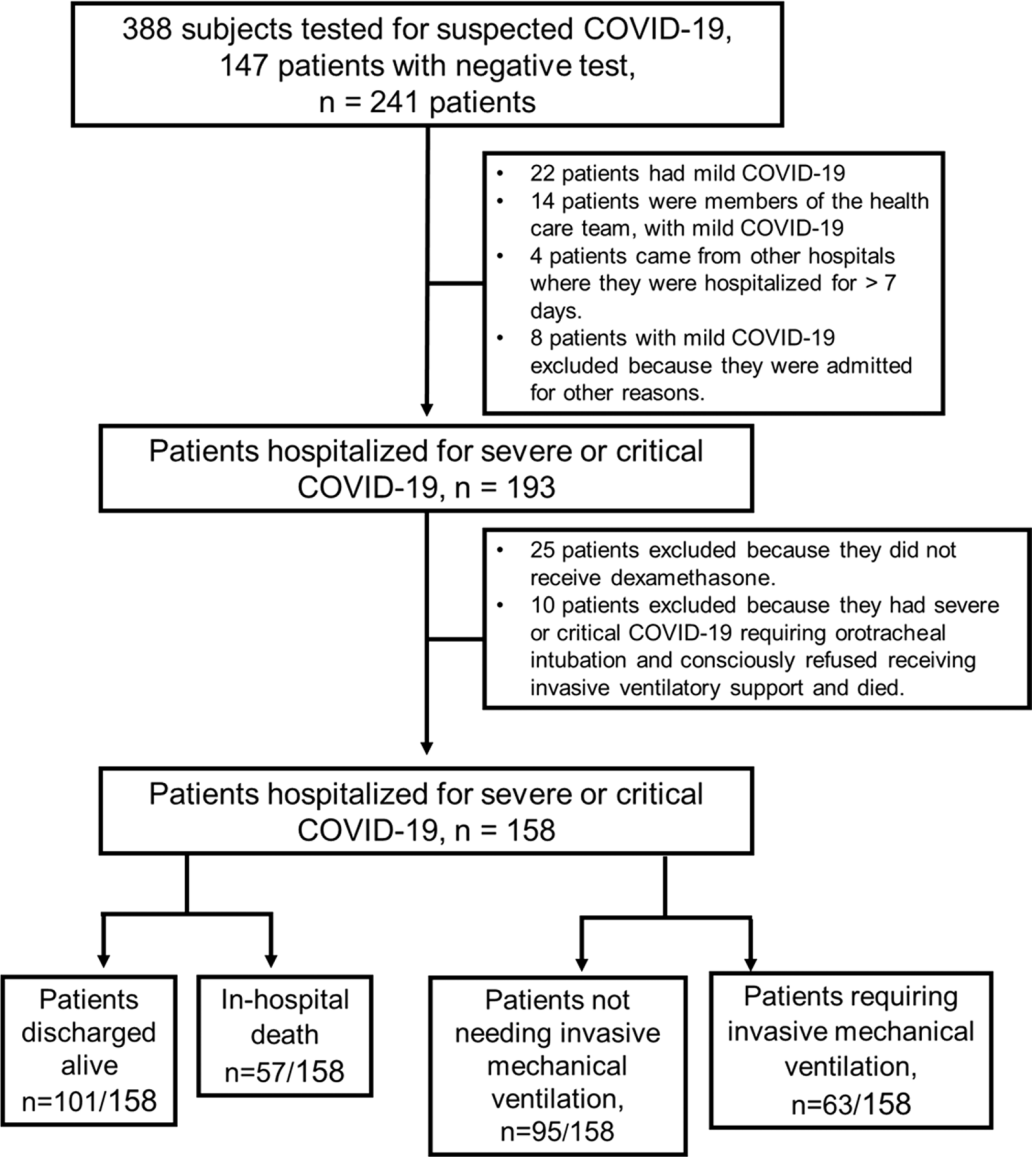
Flow chart for patient selection

A total of 158 patients were included. Figure 1 shows the flow chart for patient selection. The diagnosis was confirmed by RT-PCR in a respiratory sample in 92.4% and by an antigen test in a respiratory sample in 7.5%. The median age was 63 years old (IQR 55–75), 70% (n = 112) were men and 37% (n = 60) came from rural areas. In-hospital mortality was 36% (n = 57) and 39% (n = 63) of the patients required IMV. The most common comorbidities were high blood pressure (60%), type 2 diabetes mellitus (48%), obesity (26%) and COPD (16%). There were no differences between groups in the use of ACEI and ARB. Some comorbidities such as COPD and stage 3 and 4 chronic kidney disease (CKD) were more common in the in-hospital death group. The most common symptoms were dyspnea, cough, fever, fatigue, and myalgia. Neurological and gastrointestinal symptoms were less common. The median duration of symptoms was 15 days (IQR 13–20). The population studied was quite morbid, the median Charlson comorbidity index was 3 points (IQR 1–4). Also, the clinical severity assessment scales yielded very high scores, to mention, the median NEWS2 score was 7 points (IQR 5–9) and the median PSI/PORT score was 90 points (IQR 70–112); the PSI/PORT score was > 90 points in 49% and APACHE II score was ≥ 10 points in 59%. Table 1 summarizes the demographic and clinical characteristics of the study groups. See Supplementary Table 1 for all the clinical and laboratory abnormalities.

**Table 1:** Demographic and clinical characteristics

Variable	n = 158 (%)	Patients discharged alive n = 101(%)	In-hospital death n = 57(%)	p	Patients not needing invasive mechanical ventilation, n = 95(%)	Patients requiring invasive mechanical ventilation, n = 63(%)	p
Age, mean, SD;	63.8 (29 to 96), SD 14.6	61 (29 to 96), SD 14.4	68.8 (37 to 93), SD12.4	0.00	63.2 (29 to 96), SD 16.2	64.6 (37 to 91), SD 10.7	0.048
> 60 years old	94 (59.5)	51 (50.5)	43 (75.4)	0.002	54 (56.8)	40 (63.5)	0.4
Male	112 (70.9)	70 (69.3)	42 (73.7)	0.56	66 (69)	46 (73)	0.6
Days of hospital stay, median (IQR)	9 (5–15)	8 (5.5–14)	10 (4–17)	0.8	7 (5–12)	14 (6–22)	0.005
In-hospital death	57 (36)				9 (9)	48 (76)	0.001
ICU admission	104 (65.8)	51 (50.5)	53 (93)	0.001	41 (43)	63 (100)	
ICU days, median (IQR)	9 (4–15)	8 (4–14)	11 (4–16.5)	0.5	6 (3.5–9)	12 (5–17)	0.004
Acute kidney injury	57 (36.1)	18 (17.8)	39 (68.4)	0.001	16 (16)	41 (65)	0.001
Shock requiring vasoactive support	61 (38.6)	12 (11.9)	49 (86)	0.001	2 (2)	59 (93)	0.001
High blood pressure	95 (60)	56 (55)	39 (68)	0.11	52 (54.7)	43 (68.3)	0.08
Diabetes mellitus type 2	77 (48.7)	45 (44)	32 (56)	0.16	42 (44)	35 (55)	0.1
Obesity	42 (26.6)	27 (26)	15 (26)	0.95	21 (22.1)	21 (33.3)	0.11
COPD	26 (16.5)	10 (9.9)	16 (28)	0.003	12 (12.6)	14 (22.2)	0.11
Stage 3 and 4 CKD	12 (7.6)	4 (4)	8 (14)	0.029	5 (5.3)	7 (11.1)	0.22
Current or former smoking	35 (22)	17 (16.8)	18 (31.6)	0.032	17 (17.9)	18 (28.6)	0.11

All patients met criteria for severe COVID-19. A 65% (n = 104/158) were admitted in intensive care based on clinical criteria, prognosis and critical care capacity. Length of stay for patients with IMV was longer compared to those without IMV (14 days, IQR 6–22 vs 7 days, IQR 5–12; p = 0.005). Organ dysfunction was quite prevalent, 36% had acute kidney injury. AKI presented in the second week of the disease (median of 9 days, IQR 5.5–13) and 26% of AKI-patients required conventional hemodialysis or CVVHDF. Those who died were more likely to be affected by acute kidney injury (68% vs 17%, p = 0.001). Septic shock requiring vasopressors occurred in 38% and was much more common in those who died (86% vs 11%, p = 0.001) and in those who required IMV (93% vs 2%, P 0.001). Those who died were more likely to had elevated levels of high-sensitive troponin I, total bilirubin, C-reactive protein (CRP), D-Dimer and LDH levels. A 32% had high levels of troponin I as a marker of myocardial injury and 46% had elevated levels of total bilirubin as a marker of liver dysfunction. Lymphopenia was common in all groups. Very low lymphocyte counts (below 650 cells) on CBC were observed in 74% of patients who died and 74% of those who required IMV. Ferritin levels were not associated with in-hospital death or IMV requirement.

### Pharmacological treatment and respiratory support

Table 3 summarizes the respiratory support provided. IMV was provided in 39.9% (n = 63/158) of the patients at some point during the disease. Antibiotics were use in 71% and 19% received ivermectin. All patients received dexamethasone with a proposed schedule of 10 days.

Overall, 39.8% of the population required IMV. All patients with IMV met the Berlin criteria for ARDS, and this was severe in 90.4%. All patients received a tidal volume of 8 mL/kg or less of predicted body weight, plateau pressure was measured in all patients, the median PEEP (positive end-expository pressure) was 12 cmH2O (IQR 10–12) and the use of prone positioning and neuromuscular blockade were high (68% and 77% respectively). Mortality rates for those who received IMV was 76% (n = 48); death was seen more often in elderly patients: 29% (n = 14/48) were under 60 years old, 71% (n = 34/48) were over 60 years old. Septic shock explained 66% (n = 32/48) of the deaths in IMV-patients and refractory hypoxemia explained 25% (n = 12/48) of the deaths in IMV-patients.

### Microbiological findings

Bacterial infection was suspected in 71% (n = 113/158). Respiratory tract samples (from endotracheal aspirate and orotracheal tube) were taken immediately after performing orotracheal intubation (n = 63/158). Bacterial infection was confirmed in 53% (n = 34/63). FAPP was tested in 51 patients and showed the presence of bacteria in 41% (n = 21/51). No other virus was detected by this media. In another 13 patients the conventional tracheal aspirate culture showed growth for bacteria. It should be noted that the FAPP detected ≥ 2 bacteria in 38% (n = 8/21). Putting all the results together, 53 bacteria were obtained in 34 patients. *Klebsiella pneumoniae* was found in 33.9%, *Haemophillus influenza* in 15% and *Enterobacter* spp was found in 11.3%, *Staphylococcus aureus* was found in 16.9% and *Streptococcus pneumoniae* in 7.5%. Ventilator-associated pneumonia (VAP) was later diagnosed in 23%. A total of 133 patients were tested for influenza antigen test and were all negative.

### Risk scales and prognosis

When the data collection of the study began, the specific scales for risk and prognosis of COVID-19 patients were not yet known. Here, several risk and prognostic classification scales were evaluated. People who died and people who required IMV had higher Charlson comorbidity index, NEWS2 score, MuLBSTA score, qSOFA score, PSI/PORT score, SOFA score, CURB 65 score and APACHE II score. Table 2 summarizes clinical and prognostic classification scales.

**Table 2:** Clinical and prognostic classification scales

Variable	n = 158 (%)	Patients discharged alive n = 101(%)	In-hospital death n = 57(%)	p	Patients not needing invasive mechanical ventilation, n = 95(%)	Patients requiring invasive mechanical ventilation, n = 63(%)	p
Charlson comorbidity index, median (IQR)	3 (1–4)	2 (1–3)	4 (2.5–6)	0.001	2 (1–4)	3 (2–5)	0.033
Charlson ≥ 3 points	86 (54.4)	43 (42.6)	43 (75.4)	0.001	47 (49)	39 (61)	0.12
News2 score, median (IQR)	7 (5–9)	6 (5–8)	9 (7–11)	0.001	6 (5–8)	8 (7–11)	0.001
News2 score: ≥ 7 points	92 (58.2)	46 (45.5)	46 (80.7)	0.001	39 (41)	53 (84)	0.001
MuLBSTA score, median (IQR)	9 (7–13)	9 (5.5–11)	11 (9–15)	0.001	9 (5–13)	11 (9–13)	0.001
MuLBSTA score, > 10 points	76 (48.1)	37 (36.6)	39 (68.4)	0.001	41 (43)	35 (55)	0.1
qSOFA score, 2–3 points	42 (26.6)	10 (9.9)	32 (56.1)	0.001	10 (10)	32(50)	0.001
PSI/PORT score, median (IQR)	90 (70–112)	79 (64.5–95.5)	112 (94–141)	0.001	79 (64–98)	105 (89–127)	0.001
PSI/PORT score, < 71 points	41 (25.9)	38 (37.6)	3 (5.3)	0.001	36 (37.9)	5 (7.9)	0.001
PSI/PORT score, 71–90 points	39 (24.7)	31 (30.7)	8 (14)	0.02	28 (29)	11 (17)	0.08
PSI/PORT score, 91–130 points	57 (36.1)	27 (26.7)	30 (52.6)	0.001	23 (24)	34 (54)	0.00
PSI/PORT score, > 130 points	21 (13.3)	5 (5)	16 (28.1)	0.001	8 (8)	13 (20)	0.02
APACHE II Score upon admission, median (IQR)	10 (7–14)	9 (7–13)	13 (10.5–18.5)	0.001	9 (7–13)	12 (10–18)	0.001
APACHE II score, ≥ 10 points	94 (59.5)	45 (44.6)	49 (86)	0.001	45 (47)	49 (77)	0.001
SOFA score, median (IQR)	3 (2–6)	2 (2–4)	5 (3.5–7.5)	0.001	2 (2–4)	5 (3–7)	0.001
SOFA score, ≥ 4 points	75 (47.5)	32 (31.7)	43 (75.4)	0.001	28 (29)	47 (74)	0.001
CURB 65 score, median (IQR)	2 (1–2)	1 (1–2)	2 (2–3)	0.001	1 (1–2)	2 (1–3)	0.001
CURB 65 score, 0–1 points	72 (45.6)	61 (60)	11 (19)	0.001	56 (58)	16 (25)	0.001
CURB 65 score, 2 points	56 (35.4)	34 (33.7)	22 (38.6)	0.5	33 (34)	23 (36)	0.8
CURB 65 score, 3–5 points	30 (19)	6 (5.9)	24 (42.1)	0.001	6 (6)	24 (38)	0.001

Logistic regression to establish risk factors for in-hospital death and invasive mechanical ventilation.

The multivariable logistic regression analysis included the variables with p-value of < 0.05 (see Tables 1, 2, 3 and Additional file 1: Table S1). We established that the risk factors for IMV were a NEWS2 score ≥ 7 points, CURB 65 score of 3 to 5 points, PaO_2_/FiO_2_ ratio < 70, highest CRP levels recorded > 27 mg/dL, lymphocytes in CBC on admission < 650 per/mm^3^ and PSI/PORT score of 91 to 130 points. The risk factors for in-hospital death were a troponin I levels above the normal range, acute kidney injury, COPD, MuLBSTA score > 10 points and LDH levels > 500 IU/L. Table 4 summarizes the logistic regression results.

**Table 3:** Characteristics of the respiratory support delivered

Variable	n = 158 (%)	Patients discharged alive n = 101(%)	In-hospital death n = 57(%)	p	Patients not needing invasive mechanical ventilation. n = 95(%)	Patients requiring invasive mechanical ventilation, n = 63(%)	p
High flow nasal oxygen and/or noninvasive ventilation	57 (36)	(37.6)	19 (33.3)	0.59	32 (33.7)	25 (39.7)	0.4
Noninvasive ventilation	24/57 (42)	13/38 (34)	11/19 (57.9)	0.08	11 (34)	13 (52)	0.18
High flow nasal oxygen	43/57 (75)	30/38 (78.9)	13/19 (68)	0.51	26 (81)	17 (68)	0.24
Invasive mechanical ventilation	63 (39.9)	15 (14.9)	48 (84.2)	0.001			
Days in Invasive Ventilatory Support, median (IQR)	9 (3–15)	10 (6–16)	7 (3–14.7)	0.6

**Table 4:** Logistic regression, risk factors for (a) invasive mechanical ventilation*, (b) in-hospital death**

Variables	OR	p value	95% CI
(a)			
News2 score: ≥ 7 points	3.012	0.032	1.1–8.2
CURB 65 score, 3–5 points	8.264	0.002	2.1–32.1
PaO2/FiO2 < 70	6.383	0.001	2.4–16.6
Highest CRP levels recorded, > 27 mg/dL	3.178	0.022	1.1–8.5
Lymphocytes in CBC on admission, < 650 per mm3	2.775	0.044	1.02–7.5
PSI/PORT score, 91–130 points	3.053	0.019	1.1–7.5
(b)			
Troponin I level above the normal range	5.300	0.001	1.9–14
Acute kidney injury	10.073	0.001	3.7–27
COPD	4.789	0.014	1.3–16
MuLBSTA score > 10 points	4.359	0.004	1.6–11
LDH levels > 500 IU/L	5.048	0.001	1.9–13

^a^In the logistic regression analysis, Omnibus tests of model coefficients had a p value of 0.001; Nagelkerke's R was 52.1% (0.521); the Hosmer and Lemeshow test showed a p value of p = 0.493

^b^In the logistic regression analysis, Omnibus tests of model coefficients had a p value of 0.001; Nagelkerke's R was 56.7% (0.567); the Hosmer and Lemeshow test showed a p value of p = 0.304

## Discussion

This study describes the clinical characteristics and risk factors of patients with severe and critical COVID-19, from a single center in Colombia. Older persons, men, and those with hypertension, diabetes, obesity, and COPD were highly prevalent. Our overall mortality rates were high and especially high in patients with IMV, mainly when those affected were elderly.

The many studies on COVID-19 have shown very different mortality rates [16, 17]. High mortality rates have been registered in Latin America [8]. Comorbidities and age influence the outcome of death. For the population of our study, only COPD was relevant in the outcome of in-hospital death. Some risk factors such as increased levels of D-dimer did not reach statistical significance in the logistic regression analysis. We found that acute kidney injury and myocardial injury were very common, both associated with in-hospital death. Troponin I (TnI) elevations correlate with a higher incidence of severe disease, ICU admission and death compared to patients with non-elevated TnI [18]. High levels of LDH were associated with in-hospital death, a finding that has been consistently reported elsewhere [19].

The findings of high mortality rates among ventilated patients have been reported, especially in the early pandemic and closely related to the care of the elderly population as in our study [16]. Lim et al. describe the phenomenon in a Meta-analysis of patients with COVID-19 requiring IMV, revealing that almost half of patients with COVID-19 receiving IMV died, but the case fatality ratio was higher in older patients and in early pandemic epicenters, probably explained by exhaustion of ICU resources [16]. The authors reported that for patients with an age range of 61–70 years, a mortality rate can be as high as 71.3% for patients receiving IMV [16].

The mortality rate in patients with ARDS requiring IMV was very high and exceeds what was reported in the pre-COVID-19 era [10]. For the population of our study, bacterial pneumonia was high prevalent at the time of starting IMV, being present in 53% of patients with IMV compared to what has been reported elsewhere [20, 21], also, ventilator-associated pneumonia was very common. Notably, patients with COVID-19 are more likely to be investigated for VAP and had a higher incidence of microbiologically confirmed VAP [22]. We do not know to what extent the findings of high frequency of bacterial infection in patients with IMV explain the high mortality in this group.

Our study showed how the risk and prognosis classification scales traditionally used for bacterial pneumonia and sepsis were also useful in the evaluation of patients with COVID-19.

### Limitations

This study had several limitations. First, the results were obtained retrospectively from a single center, which may limit the generalization of the results to a more extensive geographic context, however our data are in line with other reported analyses. Second, we concentrate our analysis in the data obtained during the first pandemic wave in Colombia, therefore the extrapolation of the results to other points of time may not be appropriate since the experience in critical covid-19 was under construction. Third, most of the clinical and laboratory variables were based on the first tests performed at the time of initial presentation of the patient and it was not considered whether there was variation in the laboratory variables during the evolution of the disease. Fourth, there may be factors of a social and economic nature that affected the outcomes and that could not be considered in the analysis, for example, due to social rejection and denial of the disease and the disposal of corpses due to covid-19, many people did not attend health care centers in a timely manner and consulted only when the disease was in advanced stages, however this situation has not been widely studied or documented in our region. Fifth, the population treated in our center was mainly elderly, therefore the results of our study should be interpreted with caution.

## Conclusions

This case series provides the clinical characteristics and outcomes of hospitalized patients with severe and critical COVID-19, in the city of Palmira, Colombia, during the first wave of the pandemic in Colombia. The risk factors for in-hospital death and IMV were described.

Our study showed that severe and critical COVID-19 yielded a high mortality rate in a mainly elderly Colombian population. Many patients were admitted to intensive care and required IMV. The cases of bacterial infection in mechanical ventilated patients were higher than expected. The strategies of protective ventilation and ventilation in the prone position were used in a high percentage of patients, however the outcomes were not satisfactory.

The risk factors for in-hospital death and IMV found in our study contribute to the comprehensive evaluation of patients and to making informed and end-of-life decisions in the mainly elderly population. The data provided also contributes to the characterization of the COVID-19 pandemic in Colombia as a reflection of the situation in Latin America.

## Supplementary Information

**Additional file 1: Table 1.** Clinical manifestations and laboratory abnormalities.

## Data Availability

The datasets used and/or analyzed during the current study are available from the corresponding author on reasonable request.

## Abbreviations

SARS-CoV-2: Severe acute respiratory syndrome coronavirus 2
COVID-19: Coronavirus disease 2019
LA: Latin America
ARDS: Acute respiratory distress syndrome
FAPP: FilmArray Pneumonia Panel
LDH: Lactate dehydrogenase
IMV: Invasive mechanical ventilation
COPD: Chronic obstructive pulmonary disease.
PEEP: Positive end-expository pressure
VAP: Ventilator-associated pneumonia
RT-PCR: Real-time polymerase chain reaction
CLSI: Clinical & Laboratory Standards Institute
AP: Alkaline phosphatase
ALT: Alanine transaminase
AST: Aspartate transaminase
CBC: Complete blood count
LDH: Lactate dehydrogenase
LR: Likelihood ratio
CVVHDF: Continuous venovenous hemodiafiltration
ARBs: Angiotensin II receptor blockers
ACEi: Angiotensin-converting-enzyme inhibitors
LFNC: Low flow nasal cannula
NRM: No Rebreathing Mask
PaO_2_/FiO_2_: Ratio of the partial pressure of arterial oxygen to the fraction of inspired oxygen
ELFA: Enzyme linked fluorescent assay
CRP: C-reactive protein
