# Supplementary material for: Severe and critical COVID-19 in a tertiary center in Colombia, a retrospective cross-sectional study
Source: BMC Infect Dis. 2022 Mar 12;22:247. doi: 10.1186/s12879-022-07246-0 (PMC8917827; doi:10.1186/s12879-022-07246-0)
Supplement: Supplementary file 1 — Additional file 1: Table 1. Clinical manifestations and laboratory abnormalities. [file 12879_2022_7246_MOESM1_ESM.docx]

| Supplementary Table 1. Clinical manifestations and laboratory abnormalities. | | | | | | | |
| --- | --- | --- | --- | --- | --- | --- | --- |
| Variable | n=158 (%) | Patients discharged alive  n=101(%) | In-hospital death.  n=57(%) | P | Patients not needing invasive mechanical ventilation, n=95(%) | Patients requiring invasive mechanical ventilation, n=63(%) | P |
| Dyspnea | 146 (92) | 94 (93) | 52 (91) | 0.7 |  |  |  |
| Cough | 123 (77.8) | 78 (77) | 45 (78) | 0.8 |  |  |  |
| Fever | 123 (77.8) | 76 (75) | 47 (82) | 0.29 |  |  |  |
| Fatigue | 67 (42) | 53 (52) | 14 (24) | 0.001 |  |  |  |
| Myalgia | 60 (38) | 42 (41) | 18 (31) | 0.21 |  |  |  |
| Headache | 24 (15) | 19 (18) | 5 (8) | 0.09 |  |  |  |
| Diarrhea | 25 (15) | 18 (17) | 7 (12) | 0.3 |  |  |  |
| Sore throat | 25 (15) | 18 (17) | 7 (12) | 0.3 |  |  |  |
| Sputum | 17 (10.8) | 9 (8) | 8 (14) | 0.3 |  |  |  |
| Ageusia and / or anosmia | 18 (11) | 13 (12) | 5 (8) | 0.4 |  |  |  |
| Vomiting | 5 (3) | 2 (2) | 3 (5) | 0.3 |  |  |  |
| Serum creatinine on admission, mg/dL, median (IQR) | 0.9 (0.75-1.3) | 0.89 (0.7-1.1) | 1.1 (0.8-1.5) | 0.001 | 0.88 (0.7-1.1) | 1 (0.8-1.5) | 0.005 |
| Lactate levels in serum, mmol/L, median (IQR) | 1.5 (1.3-2.1) | 1.5 (1.2-2) | 1.6 (1.3-2.4) | 0.02 | 1.6 (1.3-2.1) | 1.5 (1.2-2.3) | 0.4 |
| Lactate levels in serum, ≥2 mmol/L | 28/86 (32.3) | 14/49 (28.6) | 14/37 (37.8) | 0.36 | 14/43 (32) | 14/43 (32) | 1 |
| Total bilirubin levels, mg/dL, median (IQR) | 0.57 (0.36-0.81) | 0.5 (0.35-0.69) | 0.72 (0.38-0.98) | 0.2 | 0.52 (0.34-0.73) | 0.67 (0.39-0.90) | 0.6 |
| Total bilirubin levels, above the normal range, mg/dL | 48/103 (46.6) | 25/67 (37.3) | 23/36 (63.9) | 0.01 | 25 (41) | 23 (54) | 0.16 |
| CRP upon admission, mg/dL, median (IQR) | 13.7 (5.2-26.4) | 10.9 (3.9-24.2) | 19 (9.9-34) | 0.04 | 10.6 (3.8-22.6) | 20 (8.2-31.9) | 0.05 |
| Highest CRP levels recorded, mg/dL median (IQR) | 18.4 (8.3-29.8) | 15.7 (6.4-27) | 25.7 (13.4-34.5) | 0.01 | 14.6 (6-24.8) | 26.8 (14.9-36) | 0.002 |
| Highest CRP levels recorded, >27 mg/dL | 46/144 (31.9) | 23/94 (24.5) | 23/50 (46) | 0.008 | 18 (20) | 28 (50) | 0.00 |
| D-Dimer upon admission,, mcg/mL, median (IQR) | 1.39 (0.77-3.67) | 1.2 (0.73-2.59) | 1.8 (0.88-6.1) | 0.001 | 1.2 (0.71-2.8) | 1.6 (0.87-4.9) | 0.08 |
| LDH levels upon admission,, IU/L, median (IQR) | 439 (344-621) | 399 (323-512) | 597 (407-775) | 0.00 | 393 (321-512) | 545 (406-764) | 0.005 |
| LDH levels >500 IU/L | 61/153 (39.9) | 28/100 (28) | 33/53 (62.3) | 0.00 | 25 (26) | 36 (60) | 0.00 |
| Lymphocytes in CBC upon admission, cells per mm3, median (IQR) | 790 (480-1357) | 890 (492-1370) | 690 (440-1270) | 0.1 | 865 (470-1365) | 740 (475-1355) | 0.7 |
| Lymphocytes in CBC on admission, <650 per mm3 | 89/150 (59.3) | 51 (51.5) | 38 (74.5) | 0.007 | 45 (49) | 44 (74) | 0.002 |
| High-sensitivity Troponin I levels, ng/mL, median (IQR) | 51.3 (25.9-294) | 39 (18-111) | 74 (30-312) | 0.06 | 34.9 (17-114) | 64.4 (32-311) | 0.12 |
| High-sensitivity Troponin I levels above the normal range | 50/155 (32.3) | 19/99 (19.2) | 31/56 (55.4) | 0.001 | 18 (19) | 32 (51) | 0.00 |
| AST levels, U/L, median (IQR) | 52.9 (35.1-80.6) | 40 (28-65) | 59 (39-95) | 0.15 | 42 (29-72) | 61 (41-108) | 0.03 |
| ALT levels, U/L, median (IQR) | 42.7 (25.7-65) | 39 (23-58) | 37 (23-65) | 0.25 | 39 (23-61) | 52 (28-66) | 1 |
| Ferritin levels, ng/mL, median (IQR) | 1188 (596-2000) | 1124 (571-1939) | 1211 (826-2000) | 0.9 | 995 (488-1916) | 1453 (937-2000) | 0.4 |
| PaO2/FiO2 upon admission, median (IQR) | 108 (73-184) | 155 (88-205) | 80.5 (63-108) | 0.001 | 175 (91.2-209) | 81 (62-105) | 0.00 |
| Worst value of PaO2/FiO2 ever recorded, median (IQR) | 78 (62-131) | 92 (69-176.5) | 62.5 (54-83.5) | 0.001 | 106 (71-179.2) | 63 (56-78) | 0.00 |
| PaO2/FiO2 < 70 | 62 (39.5) | 27 (26.7) | 35 (62.5) | 0.00 | 21 (22) | 41 (65) | 0.00 |
| PaO2/FiO2 <100 | 103 (65.6) | 57 (56) | 46 (82) | 0.001 | 46 (48.9) | 57 (90.5) | 0.00 |
| PaO2/FiO2 < 135 | 120 (76.4) | 66 (65) | 54 (96) | 0.001 | 57 (60) | 63 (100) | 0.00 |
| PaO2/FiO2 <150 | 126 (80) | 72 (71) | 54 (96) | 0.001 | 63 (67) | 63 (100) | 0.00 |
